# Perceptions of sources of transmission among hospital employees infected with severe acute respiratory coronavirus 2 (SARS-CoV-2) in an urban tertiary care hospital: a qualitative study to inform future pandemic management

**DOI:** 10.1017/ash.2025.39

**Published:** 2025-03-14

**Authors:** Ziyue Luo, Daniel E. Kent, Pooja Shah, Dina Poplausky, MacKenzie Clark MacRae, Cassidy Boomsma, Jacob M. Jasper, Alysse G. Wurcel, Elena Byhoff, Alice M. Tang, Shira Doron, Ramnath Subbaraman

**Affiliations:** 1 Department of Public Health and Community Medicine, Tufts University School of Medicine, Boston, MA, USA; 2 Division of Geographic Medicine and Infectious Diseases, Tufts Medical Center, Boston, MA, USA; 3 Division of Health Science Systems, UMass Chan Medical School, Worcester, MA, USA

## Abstract

**Objective:**

Hospital employees are at risk of severe acute respiratory coronavirus 2 (SARS-CoV-2) infection from patient, coworker, and community interactions. Understanding employees’ perspectives on transmission risks may inform hospital pandemic management strategies.

**Design:**

Qualitative interviews were conducted with 23 employees to assess factors contributing to perceived transmission risks during patient, coworker, and community interactions and to elicit recommendations. Using a deductive approach, transcripts were coded to identify recurring themes.

**Setting:**

Tertiary hospital in Boston, Massachusetts.

**Participants:**

Employees with a positive SARS-CoV-2 PCR test between March 2020 and January 2021, a period before widespread vaccine availability.

**Results:**

Employees generally reported low concern about transmission risks during patient care. Most patient-related risks, including limited inpatient testing and personal protective equipment availability, were only reported during the early weeks of the pandemic, except for suboptimal masking adherence by patients. Participants reported greater perceived transmission risks from coworkers, due to limited breakroom space, suboptimal coworker masking, and perceptions of inadequate contact tracing. Perceived community risks were related to social gatherings and to household members who also had high SARS-CoV-2 infection risk because they were essential workers. Recommendations included increasing well-ventilated workspaces and breakrooms, increasing support for sick employees, and stronger hospital communication about risks from non-patient-care activities, including the importance of masking adherence with coworkers and in the community.

**Conclusions:**

To reduce transmission during future pandemics, hospitals may consider improving communication on risk reduction during coworker and community interactions. Societal investments are needed to improve hospital infrastructure (eg, better ventilation and breakroom space) and increase support for sick employees.

## Introduction

Like other essential workers, hospital employees, including but not limited to patient-facing healthcare workers, were especially susceptible to infection early in the coronavirus 2019 (COVID-19) pandemic.^1^ Ensuring employees’ safety during pandemics is crucial for sustaining healthcare systems. However, protecting employees from infection remains a challenge. Over the pandemic’s first 18 months, the World Health Organization estimated 80,000 to 180,000 healthcare workers worldwide died from COVID-19.^2^

Several quantitative studies have evaluated sources of exposure for hospital employees with severe acute respiratory syndrome coronavirus 2 (SARS-CoV-2) infection,^3–8^ including a prior study by our team of 187 employees infected with SARS-CoV-2 at a U.S. tertiary care hospital during the pandemic’s first year.^3^ We assessed employees’ exposures to people with possible or known SARS-CoV-2 infection from patient, coworker, and community interactions. Although most employees could not identify a high-risk exposure, among those who could, high-risk exposures were most frequently reported in the community (consistent with other studies^4,5,9,10^) and secondarily from coworkers. Other studies suggest coworker exposures were more common than patient exposures and more likely to lead to transmission.^11,12^ Findings of these studies suggest that—while the focus early in the pandemic was on protecting hospital employees from infection during patient care—employees were more likely to get infected from community and coworker interactions.

Understanding employees’ perspectives on exposure risks during the COVID-19 pandemic may inform hospital strategies to reduce respiratory pathogen transmission during future pandemics. However, few studies have used qualitative research to explore hospital employees’ risks during the COVID-19 pandemic.^13–15^ Most qualitative studies focused on general experiences of healthcare workers and challenges faced very early in the pandemic, such as the uncertainty surrounding a novel virus.^14,15^ Few studies explored risks from coworker or community interactions, and, to our knowledge, studies did not interview employees who were not patient-facing or who had confirmed COVID-19.

In this study, we conducted qualitative interviews with patient-facing and non-patient-facing hospital employees to identify contextual factors contributing to SARS-CoV-2 infection risk from patient, coworker, and community interactions. We purposefully sampled from individuals who responded to our quantitative survey.^3^ We elicited reflections and recommendations focusing on the two weeks before employees were diagnosed with SARS-CoV-2. Our study reflects perspectives from the pandemic’s first year, before widespread vaccine availability. Our goal is to inform interventions to reduce risks for employees during future pandemic events.

## Methods

### Study setting

Tufts Medical Center (TMC) is a tertiary hospital with about 7000 employees in Boston, Massachusetts.^3^ Universal (non-N95) masking for employees, visitors, and patients was initiated on March 27, 2020. In the pandemic’s initial months, N95 respirator use was only recommended during contact with COVID-19 patients in intensive care units or for aerosol-generating procedures, following Centers for Disease Control and Prevention (CDC) guidelines during severe shortage. N95 respirator use by providers for all interactions with patients with suspected or confirmed COVID-19 was implemented on July 11, 2020. SARS-CoV-2 PCR testing for all patients at admission was initiated on May 19, 2020.

### Research team characteristics and positionality

This study team comprised medical students (DK, DP, MCM, CB, JJ), a public health student (ZL), an undergraduate student (PS), public health faculty (RS, AMT), and attending physicians (SD, RS, AGW, EB), including one leading infection control (SD). Most team members were women (DP, MCM, CB, ZL, PS, AMT, SD, AGW, EB); the rest were men (DK, JJ, RS).

### Initial survey and participant sampling for qualitative interviews

Study participants were TMC employees with a positive SARS-CoV-2 PCR test between March 1, 2020, and January 15, 2021. The survey’s methods were published previously.^3^ Of 573 TMC employees who tested positive for SARS-CoV-2, 187/573 (32%) participated in this survey, of whom 166/187 (89%) were willing to be contacted again and could be recruited for qualitative interviews.

Starting in July 2022, we recruited qualitative interview participants using purposeful sampling. To achieve a diverse sample, we categorized eligible participants by gender, race, ethnicity, and job type. Using criteria to achieve representation across characteristics, we contacted potential participants in groups of five by email and then a single phone call and voicemail if a person did not respond. Due to limited responses, recruitment was expanded to include all employees who had indicated willingness to be contact again in the survey. We stopped recruitment after completing 23 interviews, as initial analysis suggested thematic saturation.

### Data collection

One-time interviews with each participant were conducted by authors DK and ZL between July and November 2022 using Zoom video-conferencing software. Each interview lasted 20-45 minutes and was recorded. As interviewers had not worked in the hospital, they were not previously known to participants. Zoom performed interview transcription. Interviewers corrected transcripts against the audio files and uploaded de-identified transcripts into Dedoose software. Transcripts were not returned to participants for verification.

Interview guides followed a framework eliciting information on perceived infection risks across the three settings of interest, and recommendations for hospital interventions to reduce risk. The period participants were asked to reflect upon was the two weeks before their SARS-CoV-2 diagnosis. As such, interview findings reflect experiences from the pandemic’s initial year, before vaccines or outpatient antiviral therapy were widely available and when hospitals were adapting to new information.

The interview guide started with open-ended questions asking participants how they believed they were infected (Supplementary Text). Subsequent sections asked open-ended and semi-structured questions about risks from patient, coworker, and community interactions, aligned with the framework of our prior survey and other studies.^3–5^ The interview closed with questions about the hospital’s response and elicited recommendations.

### Data analysis

We used a deductive approach to thematic analysis. A coding framework was developed based on quantitative survey findings and the interview guide structure (Supplementary Table). The framework was refined using post-interview impressions by interviewers DK and ZL. Code categories aligned with settings of transmission—ie, via community, patient, or coworker interactions—and a fourth category captured recommendations. Within each category, codes addressed setting-specific challenges—eg, patient masking codes for the patient setting, contact tracing codes for the coworker setting.

Each transcript was coded independently by two researchers (among DK, ZL, PS, DP, MM, and CB) using Dedoose software.^16^ Inter-rater reliability was not assessed, as coding was reconciled for all transcripts through discussion. During initial coding, new codes were occasionally incorporated into the coding scheme. Transcripts were coded a second time to apply new codes. After completing coding, we identified recurring themes that were common or salient (ie, reported by a few participants, but that were thought to be important). Themes provided insights on factors contributing to SARS-CoV-2 exposure risk or informed recommendations for future hospital interventions. Factors represented a combination of exposures participants perceived as having contributed their own infection event and general perceived infection risks within each setting. Themes were refined through team discussions, and representative quotations were identified to illustrate each theme. Findings were not disseminated to participants.

### Ethical approval

This study was approved by the Tufts Health Science Institutional Review Board. Participants signed an electronic consent, and verbal consent for audio recording was obtained.

## Results

### Participant characteristics

The median age of the 23 participants interviewed was 36 years (interquartile range 23 to 54 years). Most were women, of white race and non-Hispanic ethnicity, and had patient-facing jobs (Table 1).

**Table 1.** Demographic characteristics of the interview participants

Characteristics	Participants (N = 23)
	No. (%)
**Gender**	
Male	5 (22)
Female	17 (74)
Prefer not to answer	1 (4)
**Race**	
White	20 (87)
Asian	1 (4)
African American	1 (4)
Other	1 (4)
**Ethnicity**	
Non-Hispanic	20 (87)
Hispanic	1 (4)
Prefer not to answer	2 (9)
**Employment**	
Patient facing	21 (91)
Non-patient facing	2 (9)
**Job titles**	
Registered nurse	7 (30)
Resident or fellow physician	3 (13)
Technician	2 (9)
Respiratory therapist	2 (9)
Pharmacist	2 (9)
Nurse practitioner	1 (4)
Physical therapist	1 (4)
Medical assistant	1 (4)
Nutrition service worker	1 (4)
Other patient-facing position	1 (4)
Administration (non-patient-facing)	2 (9)

### Patient interactions—perceived infection risks

One recurring theme indicated low perceived infection risk with patient interactions during the pandemic’s first year, reflecting a sense of strong hospital support to ensure safety during patient care. For example, the following quotation reflects a theme of adequate personal protective equipment (PPE) availability during patient interactions that was expressed broadly by participants:

*“There was great PPE* [availability] *at the hospital! I felt very safe. I felt covered*.” (Registered nurse 1)

Participants also described a few challenges contributing to risk during patient interactions that were more relevant in the pandemic’s earliest weeks. Participants often acknowledged that these limitations resulted from evolving knowledge about COVID-19 or societal challenges (eg, supply chain deficits) experienced across U.S. hospitals.

For example, during the pandemic’s initial weeks, patient-facing employees did not use masks for patients without suspected or confirmed COVID-19, due to lack of a universal mandate for providers and patients, which contributed to perceived infection risk (Table 2, Q1). Participants reported some difficulties with mask availability (Table 2, Q2).

**Table 2.** Representative quotations on contextual factors contributing to perceived risk of SARS-CoV-2 infection during patient, coworker, and community interactions

Factor contributing to perceived infection risk	Quotations
**Patient interactions**	
*Masking*: Lack of a universal masking policy or lack of availability of masks^a^	Q1. “In my opinion, [I got infected by a] patient I had cared for [in the first few weeks of the pandemic], because neither of us were wearing masks. I was close with her putting in the IV [intravenous catheter] and helping her to the bathroom, so I felt confident that was the source of my COVID[-19]…the huge thing was just lack of knowledge at the time.” (Registered nurse 2)
	Q2. “It was hard to find them [masks], especially in the beginning. Eventually they were everywhere…” (Resident or fellow physician 1)
*Screening*: Challenges with testing patients for SARS-CoV-2 infection^a^	Q3. “It took time to find out if the patient was positive [to place them under enhanced infection precautions], so the patient could be positive for days and days before we ever had a [SARS-CoV-2] test, because the test [availability] in the beginning was so scarce.” (Registered nurse 3)
	Q4. “I’m pretty convinced I got it from [a patient who] had been reporting symptoms that sounded very COVID-like, and they didn’t test him until the day after they consulted us [to see the patient]…I was in there for an hour and a half, and then the next day they’re like, ‘Oh, he has these symptoms, you should probably test for COVID.’ So yeah, [I wish they had] better testing in the beginning.” (Resident or fellow physician 1)
*Masking*: Suboptimal adherence to masking by patients	Q5. “Even COVID-positive patients, you’re not going to have someone put on a mask when they’re struggling to breathe.” (Physical therapist)
	Q6. “You know our patients just refuse to wear masks in the emergency room on the unit, and it’s hard [with] someone [who is] psychotic to force them to wear a mask…” (Resident or fellow physician 1)
**Coworker interactions**	
*Masking*: Suboptimal adherence to masking when with coworkers	Q7. “If you’re in [the office or charting room] with one person you usually end up taking off your masks just because it’s kind of exhausting to wear it all day.” (Resident or fellow physician 1)
	Q8. “The offices are pretty small. So, when you go in to talk to your coworkers, there is no six feet [physical distancing] … [and] it was just, very, very difficult to keep those [masks] on at all times…So, you would take them off around your coworkers because you felt safe.” (Medical assistant)
*Breakrooms and office spaces*: Limited and poorly ventilated breakrooms and office spaces for physical distancing	Q9. “[T]he biggest risk, that I saw, was more so having to try to do our work in an office that you shared with, you know, three or four other [healthcare providers], who are all in the office trying to eat, make phone calls and do the same things that you do.” (Nurse practitioner)
	Q10. “You’re still eating with people… We tried in our breakrooms to separate people each six feet, if we could, but most breakrooms are small…the breakrooms probably should have been individual…But how do you do that in the hospital with three thousand employees? You don’t have three thousand spare rooms.” (Respiratory Therapist 1)
*Contact tracing*: Perceived challenges with contact tracing and notification of exposure	Q11. “[A colleague] got tested, and she was positive…she stayed out of work for a week. Unfortunately, I didn’t know I had been exposed…when she returned…[S]he said, ‘Why are you at work? Are you OK?’ I said, ‘What are you talking about?’…She said, ‘[T]hat patient we had…tested positive for COVID. You need to go get tested.’” (Registered nurse 2)
	Q12. “I couldn’t really detect a consistent contact tracing protocol, and I think that would be really hard to do in an environment like a hospital where you have a lot of coworkers [as contacts]. A lot of it was just word of mouth. I found out, because the person…taking care of that patient just texted me and said, ‘Oh, well, Mr. So and So has Covid,’ but it wasn’t like a formalized notification.” (Resident or fellow physician 2)
**Community interactions**	
*Household members*: Perceived risk from household members who were also essential workers	Q13. “My wife also…[was] work[ing] in a COVID unit at that time in December 2020. And as I am a respiratory therapist, you know all my patients have COVID.” (Respiratory therapist 2)
	Q14. “I got COVID not from the hospital but from my son and his girlfriend and her family [who] worked in a grocery store, and they all tested positive for COVID a week prior to me.” (Registered nurse 1)
*Small private gatherings*: Exposure during small family or private gatherings despite initial lower perceived risk	Q15. “I got infected with COVID around Thanksgiving time…[I was] just gathering with a small group of family members, for my mom’s wishes, even though we all knew this was going to happen. So*…”* (Technician)
	Q16. “It was right before Christmas. So, we were with family, a big family. Someone had COVID. We found out two days later they had COVID, and then I got COVID…[We were] not wearing masks around family members.” (Registered nurse 4)
*Stores and public transportation*: Perceived risks from stores or public transportation, often without other clear exposures	Q17. “I might have gotten it at the grocery store. I wasn’t wearing a mask at the grocery store. I might have gotten it walking through the ED [emergency department]. I don’t really know for sure.” (Administrative position)

Note. COVID, coronavirus disease; PPE, personal protective equipment; SARS-CoV-2, severe acute respiratory coronavirus 2.

a These challenges were reported only in the earliest weeks of the pandemic.

Participants described risks from lack of asymptomatic testing of patients at hospital admission and lack of confirmatory testing for patients with suspected COVID-19, due to test shortages in the pandemic’s initial weeks. This contributed to perceived risk by delaying placement of patients under enhanced infection precautions (Table 2, Q3 and Q4).

After universal masking of employees and patients became mandatory, suboptimal masking by patients contributed to perceived risk during patient interactions. This challenge was accentuated by patients’ medical conditions, which sometimes made masking prohibitive (Table 2, Q5 and Q6).

### Coworker interactions—perceived infection risks

Multiple factors contributed to perceived infection risk during coworker interactions, including suboptimal masking with coworkers, inadequate breakroom space, and perceptions of contact tracing challenges. After the universal masking mandate, participants reported they were more likely to take their masks off around coworkers, due to “exhaustion” with mask-wearing or because they felt “safer” around coworkers (Table 2, Q7 and Q8).

Difficulties with coworker masking were compounded by limited rooms for physical distancing, especially while eating or drinking. Shared offices and breakrooms had limited space and suboptimal ventilation, which were recognized as structural challenges (Table 2, Q9 and Q10).

Participants reported perceived challenges with contact tracing, including sometimes not being contacted by the hospital to inform them that a contact (whether coworker or patient) had tested positive for SARS-CoV-2. Participants sometimes learned of a contact’s diagnosis by word of mouth, though they recognized the difficulties in implementing contact tracing, especially early in the pandemic (Table 2, Q11 and Q12).

In contrast, while universal asymptomatic testing of employees was not performed, consistent with policies in nearly all U.S. hospitals, participants felt the hospital made symptomatic testing free and easily accessible, which reduced perceived risk:

“*They made the* [SARS-COV-2] *test available for employees who were considered exposed or had symptoms. It didn’t take me very long to get a test at all. It was very good, very good.*”

(Respiratory therapist 1)

### Community interactions—perceived infection risks

Perceived community risks related to other household members, small private gatherings, public transportation, and shopping. Household risks often related to household members (eg, family, roommates) who were also essential workers (eg, grocery store workers, hospital employees) and who therefore could not engage in physical distancing (Table 2, Q13 and Q14).

Participants reported exposures during small private gatherings, because of lower perceived risks with family or friends and social pressure to not engage in masking or physical distancing. Holidays were perceived as periods of increased transmission risk (Table 2, Q15 and Q16). A theme emerged of participants identifying public spaces, such as stores or public transportation, as potential sources of transmission risk, in the absence of another clear source of exposure (Table 2, Q17).

### Recommendations for reducing infection risk among hospital employees

We organize recommendations into: (1) enhanced education and guidance by hospitals, and (2) structural changes requiring societal investment (Table 3). Participants emphasized the importance of education on the scientific rationale for masking (Table 3, Q18 and 19). Sustaining masking adherence as pandemic measures extended for several months was challenging. Participants encouraged cycles of education to reinforce mask use over time (Table 3, Q20 and Q21). Participants recognized the importance of transmission in non-patient-care settings (Table 3, Q22), and recommended enhanced education and support to promote behavior change around physical distancing (eg, caution around private gatherings) and masking (eg, education on community masking), as well as provision of masks to employees for use in community settings.

**Table 3.** Representative quotations on recommendations for reducing SARS-CoV-2 infection risk among hospital employees

Recommendation	Quotations
**Enhanced education and guidance**	
*Masking*: Provision of education on the scientific rationale for masking	Q18. “[It would be helpful] [i[f there was a video that you were required to watch [on the scientific rationale for masking]…I’m a respiratory therapist. I understand the route of the aerosol [transmission of SARS-CoV-2], but someone like in housekeeping might not. They are forced to wear the mask, but they might not have the understanding of why that mask is there, and how it’s protecting them.” (Respiratory therapist 1)
	Q19. “If we have another outbreak…We need masks. Lots and lots of masks, and I think it would be helpful if there was like a public service announcement on how to wear the masks, and why the masks are effective*.”* (Respiratory therapist 1)
*Masking:* Provision of educational reinforcement to promote high adherence to masking over long time periods	Q20. “[I] guess, you know, [I would recommend education] not to continue to have us let down our guard and take our mask off…” (Nurse practitioner)
	Q21. “Provide patient education around mask usage. Even though now it’s a different ballgame…a year and a half, two years later [after the start of the pandemic]. There’s a lot of mask fatigue.” (Other patient-facing position)
*Masking and risk perception*: Education on the importance of community and coworker transmission and encouraging and supporting masking in these settings	Q22. “I just kept picturing [people’s family members] in my head. It’s a matter of respect to wash your hands, wear your mask. You could be affecting someone’s loved one, and it’s not just [about] your immediate coworkers. It’s someone’s grandmother, someone’s husband, someone you know – a lot of people at home.” (Registered nurse 5)
**Structural changes**	
*Increased space and improved ventilation in hospitals*: Need for increased space, including more breakrooms, office spaces, and individual patient rooms, as well as improved ventilation	Q23. “[Y]eah there just needs to be more space it’s just too congested … I understand it’s a business, and I think everyone gets that… But there has to be a way to maximize the benefit of bringing in money and keeping people safe at the same time.” [Pharmacist]
	Q24. “Eating is pretty high risk, just because you have to remove your mask. So, [I would recommend] making sure that there’s adequate space and time for everyone to eat.” (Resident or fellow physician 2)
	Q25. *“*I know it’s not realistic, but patients should probably have their own rooms.” (Registered nurse 5)
*Support for sick employees*: Provision of paid sick leave to increase support and enhance reporting of illnesses by employees	Q26. “As someone who had COVID…when I had it, I had to go through all of my PTO [paid time off].” (Physical therapist)
	Q27. “[The manager said,] ‘Oh, are you really sick or [are] you just calling out because you want the day off?’…They would have to break that stigma down around calling out [sick].” (Registered nurse 5)

Note. COVID, coronavirus disease; SARS-CoV-2, severe acute respiratory coronavirus 2.

Regarding structural challenges, recommendations focused on increasing space for physical distancing and improving ventilation in breakrooms, offices, and patient rooms. Participants recommended providing more spaces for employees to eat, especially when outdoor spaces are inaccessible due to weather (Table 3, Q23 and Q24). Participants noted that increasing the number of patient rooms might decrease transmission by reducing the need for patients to share rooms (Table 3, Q25).

Finally, participants felt that support for employees who got sick with COVID-19 was insufficient, particularly paid sick leave. Participants described using up paid time off days when they had to isolate due to COVID-19. Others noted that lack of paid sick leave and hospital staffing difficulties created “stigma” around calling in sick, which could increase transmission by creating social pressure to come to work when unwell (Table 3, Q26 and Q27).

## Discussion

In this study, we identified factors contributing to SARS-CoV-2 infection risk among employees in a U.S. tertiary hospital, across patient, coworker, and community interactions. Consistent with our prior quantitative study^3^ and other studies^4–7,9,10^—which found most high-risk SARS-CoV-2 exposures happened during community and coworker (rather than patient) interactions—participants reported low perceived risk with patient interactions. This perception was shaped by high levels of hospital education, support, and PPE provision for patient care. With the exception of suboptimal masking adherence by patients, other patient care risks were largely described during the pandemic’s early weeks, when the U.S. experienced nationwide testing and PPE shortages and when COVID-19 knowledge was rapidly evolving. These findings highlight the importance of national strategies to ensure testing and PPE availability to protect employees during the initial phase of future pandemics.^17,18^

In contrast to patient care settings, participants described ongoing risks during coworker and community interactions throughout the pandemic’s first year. A cross-cutting theme was the perception that interactions with coworkers or during private gatherings were more “safe” than patient care interactions, despite the fact that hospital employees may be more likely to get infected in non-patient care settings.^19–22^ Participants’ recommendations on education about masking directly aligned with these findings. Participants suggested that hospitals should educate employees about risks during coworker and community interactions, emphasize the importance of masking during non-patient interactions, provide high-quality masks for employees to use in the community, and sustain masking education over time. Notably, CDC guidelines on SARS-CoV-2 infection control in healthcare facilities have had limited guidance on provision of education or other strategies (eg, mask provision) hospitals should implement to protect employees in community settings, beyond those articulated in community guidelines for the general public.^23–25^ Guidelines during future pandemics should have an expanded focus on mitigating risks to healthcare providers from coworker and community interactions.

Perceptions of infection risk during coworker interactions were shaped by structural challenges. In many U.S. cities, hospital infrastructure was built decades ago, resulting in employees having poorly ventilated breakrooms and offices with limited space for physical distancing, which may be key sites of transmission during pandemic and endemic phases. Employees would benefit from additional spaces for taking breaks to eat and from improved ventilation systems. However, hospitals are limited in their ability to rapidly adapt infrastructure to mitigate respiratory pathogen transmission in the midst of a pandemic.^26^ Long-term societal investments are needed to retrofit existing hospitals and build future hospitals that are designed to reduced respiratory pathogen transmission.^27^ Such hospital designs should aim to increase the number of single-occupancy patient rooms and provide more well-ventilated breakroom and office space.

Finally, the need for a better culture around taking time off from work was a recurring theme. Paid sick leave was a structural intervention recommended by participants that could improve employee health, the employee experience, and infection control, by making employees more comfortable to stay home when sick.^28–30^ Participants experienced financial difficulties from taking off the required number of days for COVID-19 isolation. Workplace culture placed additional stigma on taking days off when patient volume was high. As many U.S. healthcare personnel do not have paid sick leave,^31^ stronger regulations and societal investment may support hospitals in providing this benefit, which can financially benefit hospitals as well.^32^ Future studies should explore the role of presenteeism (ie, coming to work despite being sick) in contributing to transmission risk among hospital employees.

Strengths of this study include a focus on the COVID-19 pandemic’s first year (ie, before availability of vaccines and outpatient antiviral therapies), which enhances relevance of study findings for future pandemics. We also interviewed employees who had been diagnosed with SARS-CoV-2. Our interview guide had participants focus on the two weeks before their diagnosis, such that findings are more likely to reflect exposures that may have contributed to infection. Our findings add to the limited qualitative literature on experiences of the COVID-19 pandemic by hospital employees.

Study limitations include the fact that we collected interviews in 2022, such that findings may be limited by participant recall, potentially influencing details or the accuracy of reported risk perceptions and exposure sources. In addition, while our sample size is reasonable for a qualitative study, we may not have achieved thematic saturation for findings from employee subgroups (eg, based on their job role). Despite attempts to ensure a diverse sample reflecting the broader gender, racial, and ethnic composition of hospital employees, we were limited by those who responded from our recruitment sampling frame, which mostly comprised women (74%) and white non-Hispanic employees (64%).^3^ Notably, however, most TMC employees, as in other U.S. hospitals, are women. Finally, our sample did not include physicians who were not in training and had few non-patient-facing employees, which limits the external validity of our findings.

This qualitative study provides insights into perceived infection risks faced by hospital employees during the COVID-19 pandemic’s initial year, while eliciting recommendations that could inform hospital interventions during future pandemics. Perceived patient care risks were largely limited to the pandemic’s initial weeks, whereas coworker and community risks played a more substantial role throughout the first year. Hospital employees recommended provision of sustained education on mask use during future pandemics, with a focus on mitigating risk with coworkers and in community settings. Employees also emphasized the importance of structural challenges that require broader societal investment to improve future hospital design (eg, to increase space and ventilation), while enhancing regulations and supporting hospitals to expand paid sick leave. By listening to the voices of employees, hospitals may be better prepared to reduce their risk of infection and enhance employee well-being during future pandemics.

## Supporting information

Luo et al. supplementary material 1 Luo et al. supplementary material

Luo et al. supplementary material 2 Luo et al. supplementary material

Luo et al. supplementary material 3 Luo et al. supplementary material

## Supplementary material

The supplementary material for this article can be found at https://doi.org/10.1017/ash.2025.39
