# Supplementary material for: Perceptions of sources of transmission among hospital employees infected with severe acute respiratory coronavirus 2 (SARS-CoV-2) in an urban tertiary care hospital: a qualitative study to inform future pandemic management
Source: Antimicrob Steward Healthc Epidemiol. 2025 Mar 14;5(1):e78. doi: 10.1017/ash.2025.39 (PMC11920918; doi:10.1017/ash.2025.39)
Supplement: Luo et al. supplementary material 2 — Luo et al. supplementary material [file S2732494X25000397sup002.docx]

**Supplementary Table.** Coding Scheme for Study Evaluating Perceptions of Sources of SARS

CoV-2 Transmission Among Hospital Employees

| Category | Parent Nodes | Child Nodes |
| --- | --- | --- |
| Settings of Potential Transmission Risk | Community Interactions | Private gatherings |
|  |  | Lack of COVID-19 knowledge in the community |
|  |  | Transportation |
|  |  | Household members |
|  |  | Restaurants |
|  |  | School and childcare |
|  |  | Stores |
|  |  | Other places of work |
|  |  | Other community risk |
|  | Coworker interactions | Offices |
|  |  | Coworker masking |
|  |  | Inadequate testing |
|  |  | Breakrooms |
|  |  | Self-masking while with coworkers |
|  |  | Rounding |
|  |  | Other challenges with coworker interactions |
|  | Patient interactions | Lack of supplies |
|  |  | Other PPE (i.e., other than masks) |
|  |  | Lack of COVID knowledge in the hospital |
|  |  | Masking by patients |
|  |  | Self-masking when with patients |
|  |  | COVID policies (in hospital in relation to patient care) |
|  |  | Contact tracing |
|  |  | Sanitation (i.e., cleaning of hospital rooms, etc.) |
|  |  | Screening patients |
|  |  | Other challenges with patient interactions |
| Recommendations and perspectives on the hospital response | Recommendations for reducing infection risk among hospital employees | Visitors |
|  |  | Minimizing community contacts |
|  |  | Contact tracing |
|  |  | Education on COVID-19 by the hospital |
|  |  | Hospital policies on vaccination |
|  |  | Hospital support to decease risk in the community |
|  |  | Isolation or quarantine guidelines |
|  |  | Masking guidelines in the community |
|  |  | Improving sanitation procedures |
|  |  | PPE for non-patients |
|  |  | PPE for patients |
|  |  | Public transportation |
|  |  | Increase support for sick employees |
|  |  | Increase testing |
|  |  | Improve workspaces |
|  |  | Other recommendations for reducing infection risk |
|  | Perspectives on the hospital response | Masking (positive statements only, concerns about masking are included in each risk category above) |
|  |  | Communication by, or information from, hospital leadership |
|  |  | Contact tracing |
|  |  | Employee health |
|  |  | Hospital’s COVID policies |
|  |  | Hospital support |
|  |  | Hospital leadership |
|  |  | Testing |
|  | Changes attitudes by hospital employees towards COVID-19 over time | PPE non-patient access and policies |
|  |  | Access to testing |
|  |  | PPE |
|  |  | COVID policies |
|  |  | Paying less attention to COVID |
|  |  | Other comments on changing attitudes |

Note. COVID, coronavirus disease 2019; PPE, personal protective equipment.
