# Supplementary material for: Perceptions of sources of transmission among hospital employees infected with severe acute respiratory coronavirus 2 (SARS-CoV-2) in an urban tertiary care hospital: a qualitative study to inform future pandemic management
Source: Antimicrob Steward Healthc Epidemiol. 2025 Mar 14;5(1):e78. doi: 10.1017/ash.2025.39 (PMC11920918; doi:10.1017/ash.2025.39)
Supplement: Luo et al. supplementary material 3 — Luo et al. supplementary material [file S2732494X25000397sup003.docx]

**Supplementary Text.** Interview Guide for Study Evaluating Perceptions of Sources of SARS

CoV-2 Transmission Among Hospital Employees

**Opening of interview:**

*Interviewer:* Thank you for participating in our COVID-19 survey last year, which asked questions regarding where you might have gotten infected with COVID-19 in 2020 or early 2021. At the end of that survey, you had answered a question suggesting interest in participating in a follow-up interview.

The purpose of this interview is to get more details about the circumstances you faced outside of the hospital and inside the hospital around the time you got infected. Our goal is to understand risks faced by hospital employees, so that we can provide better information and support to reduce infections among employees in future waves of COVID-19 or other respiratory viruses. Additionally, I will ask for your recommendations regarding steps the hospital can take to reduce the risk of infection by improving conditions at work or in the community for employees.

There are no right or wrong answers to my questions. I’m simply interested in any information, thoughts, or opinions you have based on your own experiences and observations.

As a reminder, we will keep all records confidential. If our discussion about how you got infected touches upon people inside or outside the hospital who you were in contact with, we are not looking for names or any other identifying information about your contacts. We are only interested in the circumstances surrounding your infection. If you feel uncomfortable at any time, you can stop participating in the interview.

Before we begin, do you have any questions for me? [Answer any questions]

In order to make sure that we have accurate reporting of information from today’s session, we would like to record the discussion on Zoom. Zoom allows us to record both video and audio, but if you prefer, you can turn off your video and we will record audio only. I am now going to start recording this session. At the beginning I will ask you if you agree to participate in this interview and if you agree to be recorded. If you do not agree to participate, I will immediately stop the recording and end the interview. If you do not agree to be recorded, I will turn off the recording and I will take notes of our interview. If you do not want to participate, we will stop immediately. Are you ready?

With your permission, I would like to start recording now. [Turn on recording]

I am with participant [state study ID number] and I have reviewed the study procedures with them.

[Ask the participant:] Do you agree to participate in this interview?

___Yes ___No

[If above response is yes, ask the participant:] Do you agree to be audio-recorded?

___Yes ___No

[If above response is yes, ask the participant:] Do you agree to be video-recorded?

___Yes ___No

**Start Interview Questions:**

To start, I would like you to think back to when you tested positive for COVID-19 in 2020 or 2021. This was a time period before there was widespread availability of the COVID-19 vaccine, before many healthcare workers had been vaccinated for COVID-19, and when the risk of severe illness from COVID-19 was therefore much greater.

1. I would first like to ask some questions about the time leading up to your COVID infection in 2020 or 2021.

1. Could you tell me your story around the time you tested positive for COVID or when you think you got COVID?
2. Could you share any ideas you have regarding how you might have gotten infected?

2. Now, I would like to ask you about risks you may have faced in 2020 and early 2021 in 3 different settings: (1) the community setting, by which I mean any location outside of the hospital; (2) due to contact with coworkers within the hospital; and (3) due to contact with patients, if you have a role that requires you to interact with patients. Feel free to talk about risks you may have faced whether or not you experienced these risks at the time you got infected.

1. What do you feel were (general) risks you faced in the community that might increase your risk of getting infected? Again, by “community” I mean any location outside the hospital.

PROBES ONLY IF NEEDED:

For example, you could discuss any specific risks you may have faced due to the following:

1. public transport
2. restaurants and bars
3. concerts and movie theaters
4. parties and home-gatherings
5. childcare
6. risk of other family members (e.g., essential workers in the household)
7. number of family members/ roommates

1. What are your recommendations to decrease risk of infection in the community?

3. Questions about coworker interactions

1. What do you feel were circumstances that may have increased your risk of getting infected by coworkers in the hospital?

PROBES IF NEEDED:

For example, you could discuss any specific risks you may have faced due to the following:

1. Space to eat/ eating with coworkers
2. Shared work spaces?
3. PPE?
4. Testing? (regularity, accessibility, not enough testing, difficulty in getting tests, etc.)
5. Contact tracing? (Shortcomings, lack of, or benefits of etc.)
6. Hospital policies?

1. What are your recommendations to decrease risk of infection in the Hospital among coworkers?

4. Questions about patient interactions

1. Is your job patient facing? (if yes, continue; if no move to Q5)

1. What do you feel were circumstances that may have increased your risk of getting infected by patients in the hospital?

PROBES IF NEEDED:

1. PPE?
2. Identification and isolation of COVID patients?
3. Clinical Procedures?
4. Hospital Policies?
5. Contact tracing? (Shortcomings, lack of, or benefits of etc.)
6. Testing? (regularity, accessibility, not enough testing, difficulty in getting tests, etc.)

1. What are your recommendations to decrease risk of infection in the hospital when interacting with patients?

5. Management of the pandemic by the hospital

- 1. What is your overall impression about how TMC was handling the COVID pandemic when you got infected? Please feel free to share any thoughts, either positive or negative.
  2. How do you feel TMC handled contact tracing—including notification of you (if you were a contact) and notification of others who might have been in contact with you?
  3. How do you feel TMC has handled the pandemic more recently?

6. Thank you for taking the time to speak with me about your experiences. Is there anything else you would like to share that we have not talked about?
